# Systematic review finds major deficiencies in sample size methodology and reporting for stepped-wedge cluster randomised trials

**DOI:** 10.1136/bmjopen-2015-010166

**Published:** 2016-02-04

**Authors:** James Martin, Monica Taljaard, Alan Girling, Karla Hemming

**Affiliations:** 1 School of Health and Population Sciences, University of Birmingham, Birmingham, UK; 2 Clinical Epidemiology Program, Ottawa Hospital Research Institute, Ottawa, Ontario, Canada; 3 Department of Epidemiology and Community Medicine, University of Ottawa, Ottawa, Ontario, Canada

**Keywords:** randomised trial, cluster, CONSORT

## Abstract

**Background:**

Stepped-wedge cluster randomised trials (SW-CRT) are increasingly being used in health policy and services research, but unless they are conducted and reported to the highest methodological standards, they are unlikely to be useful to decision-makers. Sample size calculations for these designs require allowance for clustering, time effects and repeated measures.

**Methods:**

We carried out a methodological review of SW-CRTs up to October 2014. We assessed adherence to reporting each of the 9 sample size calculation items recommended in the 2012 extension of the CONSORT statement to cluster trials.

**Results:**

We identified 32 completed trials and 28 independent protocols published between 1987 and 2014. Of these, 45 (75%) reported a sample size calculation, with a median of 5.0 (IQR 2.5–6.0) of the 9 CONSORT items reported. Of those that reported a sample size calculation, the majority, 33 (73%), allowed for clustering, but just 15 (33%) allowed for time effects. There was a small increase in the proportions reporting a sample size calculation (from 64% before to 84% after publication of the CONSORT extension, p=0.07). The type of design (cohort or cross-sectional) was not reported clearly in the majority of studies, but cohort designs seemed to be most prevalent. Sample size calculations in cohort designs were particularly poor with only 3 out of 24 (13%) of these studies allowing for repeated measures.

**Discussion:**

The quality of reporting of sample size items in stepped-wedge trials is suboptimal. There is an urgent need for dissemination of the appropriate guidelines for reporting and methodological development to match the proliferation of the use of this design in practice. Time effects and repeated measures should be considered in all SW-CRT power calculations, and there should be clarity in reporting trials as cohort or cross-sectional designs.

**Strengths and limitations of this study**

- This is the first systematic review of stepped-wedge cluster randomised trials (SW-CRTs) to assess reporting adherence to CONSORT items.
- This high-quality systematic review has well-defined inclusion criteria, used double data abstraction throughout and clearly defines how items were classified and abstracted.
- This review identifies not only whether SW-CRTs are reporting sample size calculations, but also identifies whether the appropriate power calculation was used.
- While reporting of adherence to guidelines demonstrates quality of reporting, our review did not replicate sample size calculations.

## Background

The parallel cluster randomised trial (CRT) is commonly used in the evaluation of interventions delivered at the level of the cluster.^1–3^ In the conventional parallel CRT at the beginning of the trial, half of the clusters are randomised to the intervention and half to the control. In the stepped-wedge CRT (SW-CRT), clusters are sequentially randomised to cross from the control to intervention arm.^4–6^ Systematic reviews examining the types of interventions and breadth of use of this trial design show that while its use is still relatively rare compared with other study designs it is on the increase.^4^ ^5^ Furthermore, a recent review, focusing on the scope of interventions and rationale for the use of the design, suggests there has been a dramatic increase in the number of published SW-CRTs within the last couple of years.^7^

In parallel CRTs it is well known that sample size calculations and analysis should take into account the clustered nature of the data.^8^ ^9^ Sample size calculations which do not make allowance for this clustering underestimate the sample size needed, and analysis without adjusting for clustering leads to overly precise estimates of treatment effects. Allowance for clustering at the design stage of a parallel CRT simply requires inflation of the sample size needed under individual randomisation by the design effect for parallel cluster trials.^7^ ^8^ There are variations on this design effect for unequal cluster sizes.^10^ ^11^

**Figure 1:**
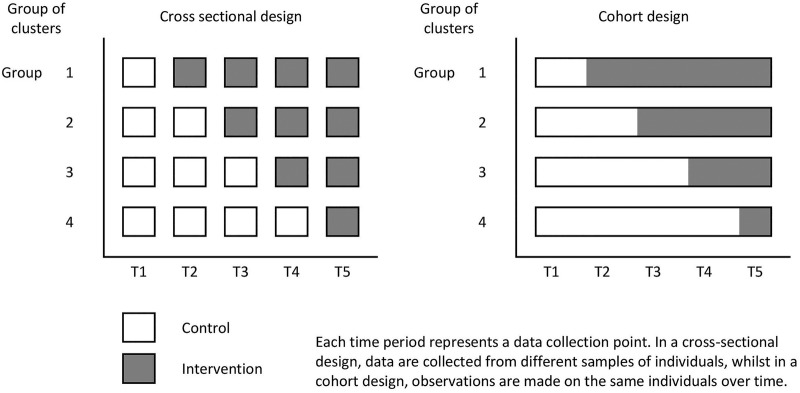
Schematic illustration of the stepped-wedge cluster randomised trial.

When using a SW-CRT, the evaluation happens over a period of time, and during this period of time, the proportion of clusters which are exposed to the intervention gradually increases. See figure 1 for an illustration of the SW-CRT. This means that the control clusters, will on average, contribute observations from an earlier calendar time than the intervention clusters. Calendar time is therefore a potential confounder and will have to be adjusted for in the analysis. Furthermore, in SW-CRTs, at each measurement occasion, the sample may be consistent of different individuals (ie, cross-sectional), or it might consist of the same individuals measured repeatedly over time (ie, cohort design). Because of this, sample size calculations for SW-CRTs should make allowance for both the clustered nature of the data and calendar time, and make an allowance for any repeated measures on the same individuals.

Hussey and Hughes^12^ first derived a method of estimating the power available from a SW-CRT which makes allowance for both the clustering and the time effects. Subsequent to this, a design effect for stepped-wedge studies has been derived, which allows determination of number of clusters needed for a given cluster size and number of steps.^13–15^ As yet, there is no adjustment to these design effects available to account for designs which involve repeated measurements on the same individuals. We do not present the design effect for the SW-CRT here as it is algebraically complicated but it can be found in the referenced papers.

Transparent reporting of clinical trials allows critical appraisal and assessment of the robustness of results.^16^ The CONSORT (consolidated standards for reporting) statement for individually randomised controlled trials (RCTs) recommends that sufficient information be provided to allow replication of the sample size calculation.^17^ ^18^ For parallel CRTs, there are additional recommendations.^19^ ^20^ Furthermore, reporting of the method used to determine the sample size allows assessment of methodological rigour. There is as yet no CONSORT extension for SW-CRTs, although one is in development^21^ and several extension items have been recommended for reporting.^6^ However, as SW-CRTs are a form of cluster RCTs, they should—as a minimum—be reported according to the CONSORT extension for CRTs.

Early CRTs were often underpowered and analysed incorrectly.^21^ Although sample size methodology and reporting guidelines for parallel CRT designs are now well established, the quality of their reporting is still assessed as being inadequate.^22–24^ But, little evidence is available on the quality of reporting, or methodological rigour, in SW-CRTs. This is because the systematic reviews of SW-CRTs to date have been small; or have not assessed adherence to reporting of the sample size calculation in accordance with CONSORT guidance; and have also not assessed whether the sample size methodology matches the design of the study.^4^ ^5^ ^7^ While it is well known that sample size reporting, particularly from studies using complex designs is poor, highlighting areas of poor performance in the SW-CRT design early on will potentially mitigate any poor practices becoming routine poor practices. It will also allow identification of items of importance to be considered for inclusion in the CONSORT extension. We have therefore undertaken a methodological review of SW-CRTs, evaluating adherence to the CONSORT cluster extension and assessment of methodological rigour of the sample size methodology used. This review forms a preparatory step in the development of the CONSORT extension for the SW-CRT, and will form one of a number of distinct bodies of work needed in the build up to this extension.^20^

Our specific objectives were to carry out a systematic review of published SW-CRTs to (1) determine adherence to reporting each of the sample size items recommended in the 2012 extension to the CONSORT statement to cluster trials; (2) identify the power methodology used in these designs; and (3) determine whether the appropriate methodology is being used, with particular emphasis on whether these trials are making allowance for both the clustered nature of the data; the time effects associated with the stepped-wedge design; and any repeated measures on the same individuals.

## Methods

### Search strategy

We used an adaptation of two previously published search strategies.^4^ ^5^ This search strategy is described in full in online supplementary figure S1, and included all protocols (not yet published as full trial reports) and independent full trial reports of SW-CRTs in both healthcare and non-healthcare settings. To meet our definition of a SW-CRT, the study had to be a randomised trial, use cluster randomisation, and have two or more steps. We excluded trials which were not published in English, individually randomised trials, trials with cross-over designs, non-randomised designs or trials which were retrospectively analysed as stepped-wedge studies. We only included original research studies and primary study reports.

We searched MEDLINE, EMBASE (including Embase classic) and PsycINFO, up to 23 October 2014. The titles and abstracts of the studies identified were screened independently by two authors (JM and one other author). Full-text articles were obtained for all potentially eligible studies and the same duplicate method of assessment used. Those found not to meet the eligibility criteria were excluded at this stage and tabulated by reason for exclusion. Any differences of opinion were resolved by discussion with all authors. We also screened reference lists of studies found to meet the inclusion criteria. We did not contact authors of papers for additional information as our primary intention was to assess quality of reporting.

We did not access published or unpublished trial protocols even if they were cited in the fully published trial report. Our motivation here was that in an assessment of reporting quality, reporting of important items (such as the sample size) should be complete in the full report and it should not be necessary to abstract information from elsewhere. To increase the available number of studies for our review, we abstracted a selection of items from study protocols which had yet to be published as completed trial reports.

### Data abstracted from trial reports

Data for all studies meeting the eligibly criteria were abstracted by two independent reviewers in random order. Any differences were resolved by consensus discussion with all authors. A data abstraction form was developed and tested on a small number of studies and then refined. Abstracted items are summarised in online supplementary table S1.

### Data abstracted on basic trial characteristics

We report the trial characteristics (for completed trials as well as subgroup of protocols only), including year of publication, country (broadly categorised into higher, lower or middle income country),^25^ journal impact factor (taken from Web of Science, JCR Science Edition 2013), type of cluster, health setting or non-health setting, number of interventions compared, whether any restriction was used in the randomisation procedure and the type of primary outcome (binary, continuous, count, etc).

For completed trials, we also summarised the design features specific to the SW-CRT, including the duration of the study, number of steps (defined as the number of randomisation points), total number of clusters, number of clusters randomised at each randomisation step, whether the design was cohort or cross-sectional, cluster size (for cohort studies this is total number of observations made across the cluster), and whether any variations on the conventional stepped-wedge design was used (eg, extended pre-period and postperiod). We also collected information on the median duration between two successive randomisation points, and the number of distinct data measurement points, which in a conventional SW-SCT is simply one greater than the number of steps. If there was a difference between planned and realised design features, we used the realised design features.

### Data abstracted on sample size reporting

For completed trials, as well as for the subgroup of trials with a protocol only, we then reported adherence to recommendations for sample size calculations as specified in the CONSORT 2010 statement,^17^ the cluster extension^18^ ^19^ and those recommended for stepped-wedge studies.^6^ Items relating to the quality of reporting of the basic sample size included reporting of: (1) the significance level, (2) the power and (3) the treatment effect; (4) whether there was consistency between primary outcome and power outcome; (5) whether or not attrition was accounted for; (6) the anticipated cluster size (or number of clusters); (7) the assumed intracluster correlation (ICC) or equivalent; (8) a measure of variation or uncertainty of the ICC; and (9) a measure of variation in cluster sizes. We deemed the treatment effect to be sufficiently reported if there was: a standardised effect size; a mean difference and SD; means in both arms and SD; proportions in both arms; proportion in one arm and an absolute or relative difference.

Elements relating to the quality of reporting of the stepped-wedge sample size included reporting of the number of steps; number of clusters randomised per step; whether a schematic representation was provided; whether there was explicit clarity over whether the design was cohort or cross-sectional; and whether there was clarity over total cluster size and cluster size per measurement point.

All items that were not clearly reported were classified as either unclear or deducible if they could be derived unambiguously from other reported items.

### Data abstracted on methodological rigour of sample size calculation

To assess methodological rigour of the power and sample size calculations, we abstracted information on how these calculations were undertaken. This information was abstracted for completed trials, as well as for the subgroup of trials with a protocol only. Of primary interest was whether the calculation adjusted for clustering, time effects and any repeated measures on the same individual. For those studies adjusting the power calculation for time effects, we determined whether the authors made reference to using the Hussey and Hughes methodology,^11^ the Woertman design effect^12^ or an alternative method which we noted. For cohort designs, we abstracted information on whether allowance was made for repeated measures on the same individual. Information was also abstracted on whether this power calculation included allowance for any transition periods;^26^ plans to explore whether the effect varies with time since exposure (ie, a learning or lag effect); or any extended correlation structures, such as an allowance for the within cluster correlation to differ for observations within different measurement periods.^27^ We also abstracted information on whether there was any allowance for varying cluster size.

### Analysis of results

We first summarise the basic trial demographics of the full trial reports and the trial protocols. We stratify this analysis by type of report (full trial report and protocol), as we expected trial protocols may be of a different demographic to the full trial report. We observed little difference between the study characteristics of the full reports and trial protocols, and so all other analyses were pooled across full trial and protocols. We then summarise the realised design characteristics of the included full trial reports.

To explore whether the publication of the CONSORT extension for cluster trials might have improved quality of reporting, we stratified by trials published before and during 2012 (the date of the publication of the cluster CONSORT extension^19^) and those published during or after 2013. While the CONSORT extension does not specifically address stepped-wedge cluster trials, stratification still allows investigation of any improvement over time. Improvements were described using absolute differences with 95% CIs. We tested these differences using a χ^2^ test for proportions, Fisher's exact test for low counts or a Mann-Whitney U test for continuous data.

## Results

**Figure 2:**
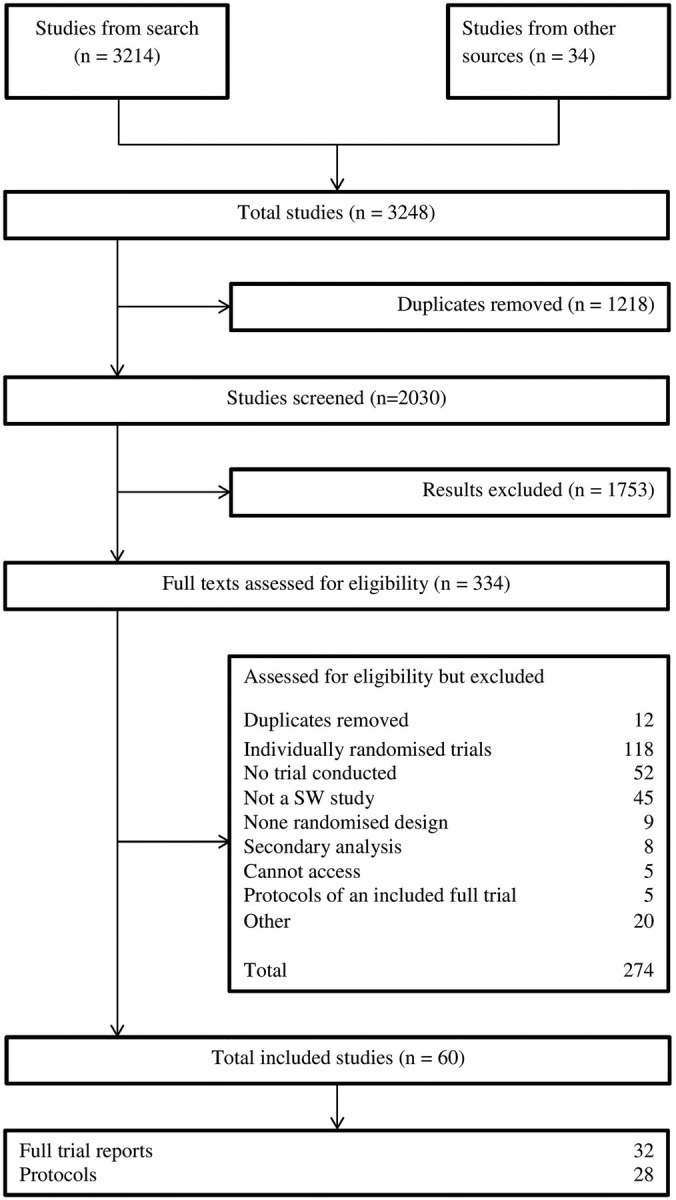
Flow chart showing studies identified by the systematic review. SW, stepped-wedge.

The searches identified 3248 studies of which 1218 were immediately identified as duplicates and 1696 were excluded on the initial abstract screen, leaving 334 full-text articles which were assessed for eligibility. Of these, we excluded 274 (details in figure 2) after a careful screen of the full paper. From this, we were left with 32 full trial reports and 28 trial protocols for inclusion in the review. A list of the included studies is provided in online supplementary appendix 1.

The trial characteristics of the 28 trial protocols and 32 full reports are summarised in table 1. Over half of both protocols and full reports were published during or after 2013. A large proportion of trials were conducted in higher income countries, and the majority (83.3%) were conducted in health settings. Examples of studies in non-healthcare settings included a study evaluating the effect of free school meals on academic attainment and an intervention to mitigate absenteeism in the workplace. Almost all studies compared two interventions (ie, standard care/control and a new intervention). The majority of studies used a simple, unrestricted form of random allocation, but a few studies used paired or stratified allocation methods. Almost 60% of the studies had a binary primary outcome, with continuous outcomes being less common.

**Table 1:** Basic trial demographics of included SW-CRTs, values are numbers (percentages) unless stated otherwise

	Total N=60	Protocols N=28	Full reports N=32
Year of publication			
1987–2012	28 (46.7)	12 (42.9)	16 (50.0)
2013–2014	32 (53.3)	16 (57.1)	16 (50.0)
Journal Impact Factor			
Median (IQR)	2.6 (2.0–3.5)	2.3 (2.1–4.8)	3.3 (2.0–4.8)
Country of study			
Australia	7 (11.7)	6 (21.4)	1 (3.1)
Canada or USA	15 (25.0)	4 (14.3)	11 (34.4)
UK or Ireland	11 (18.3)	3 (10.7)	8 (25.0)
Other higher income country	15 (25.0)	9 (32.1)	6 (18.8)
Middle-income country	9 (15.0)	4 (14.3)	5 (15.6)
Low-income country	3 (5.0)	2 (7.1)	1 (3.1)
Type of setting			
Healthcare	50 (83.3)	25 (89.3)	25 (78.1)
Non-healthcare	10 (16.7)	3 (10.7)	7 (21.9)
Cluster			
General practice	7 (11.7)	6 (21.4)	1 (3.1)
Hospital/ward/specialties	12 (20.0)	5 (17.9)	7 (21.9)
Other health cluster	20 (33.3)	9 (32.1)	11 (34.4)
Geographical unit	11 (18.3)	5 (17.9)	6 (18.8)
Other/unclear	10 (16.7)	3 (10.7)	7 (21.9)
Number of study arms			
Two	56 (93.3)	25 (89.3)	31 (96.9)
Three or more	4 (6.7)	3 (10.7)	1 (3.1)
Randomisation type			
Simple	35 (58.3)	15 (53.6)	20 (62.5)
Paired	4 (6.7)	0 (0)	4 (12.5)
Stratified	14 (23.3)	10 (35.7)	4 (12.5)
Other/unclear	7 (11.7)	3 (10.7)	4 (12.5)
Primary outcome type			
Continuous	15 (25.0)	10 (35.7)	5 (15.6)
Binary	34 (55.7)	13 (4.4)	21 (65.6)
Other	5 (8.3)	2 (7.1)	3 (9.4)
Unclear/not reported	6 (10.0)	3 (10.7)	3 (9.4)
Published protocol		NA	5 (15.6)

NA, not available; SW-CRT, stepped-wedge cluster randomised trial.

Among the 32 completed studies (table 2), the median number of randomisation steps was 4 (IQR 2–6); the median number of clusters 17 (IQR 8–38); and the median cluster size (across all measurement points) was 55 (IQR 24–326). Only 5 (15.6%) of the 32 completed studies were of cross-sectional design, with the majority being cohort (37.5%) or open cohort (31.3%) designs. Overall, 17 variations on the typical stepped-wedge design were observed, the most common of which was extended pre-period or postperiod (10 studies).

**Table 2:** Summary of the realised design features of the included stepped-wedge cluster randomised trial. Values are numbers (percentages) unless stated otherwise

	Full trial report N=32
Number of steps*	
Two	9 (28.1)
Three or four	8 (25.0)
More than four	14 (43.8)
Not reported	1 (3.1)
Median (IQR)	4.0 (2.0–6.0)
Number of clusters	
Less than 10	9 (28.1)
10 or more	22 (68.8)
Not reported	1 (3.1)
Median (IQR)	17.0 (8.0–38.0)
Total cluster size†	
Median (IQR)	55.0 (24.0–326.0)
Number of clusters randomised per step	
Median (IQR)	3.0 (1.0–8.0)
Number of measurement points‡	
Median (IQR)	5.0 (3.0–7.5)
Study duration (months), median (IQR)	16.0 (8.0–24.0)
Step duration (months), median (IQR)	2.0 (1.0–4.0)
Design type§	
Cross-sectional	5 (15.6)
Cohort	12 (37.5)
Open cohort	10 (31.3)
Unclear	5 (15.6)
Variations on design	
Transition periods	1 (3.1)
Extended pre-period or postperiod	11 (34.4)
Other	6 (18.8)

*Steps are points at which clusters are randomised.

†For cohort studies this is the total number of observations made within the cluster, it includes the size of clusters in which there was lack of clarity of cluster size and cluster size per measurement period but for which a judgement was made.

‡Measurement points are the number of separate periods or points in time in which outcome data are collected.

§Design type includes those for which there was lack of clarity but for which a judgement was made.

Overall, 45 (75.0%) of the trials reported a sample size justification (table 3). The median number of CONSORT items reported across all 60 studies was 5 (IQR 2–6). None of the studies reported all nine CONSORT items. Almost all of the studies reported the number of clusters (96.7%). Approximately, 55% of the studies reported an ICC or equivalent, but few studies reported any variation in cluster size or reported any uncertainty in the estimation of the ICC. Allowance for attrition was poorly reported with only 30.0% of studies clearly reporting this item. We observed some improvement in reporting over time, most notably, reporting of the ICC increased from 39.3% pre-2012 to 68.8% post-2013 (p=0.022).

**Table 3:** Quality of reporting of basic sample size elements from the CONSORT 2010 statement and the Cluster 2012 extension to the CONSORT statement

	All studies N=60	1987–2012 N=28	2013–2014 N=32	Absolute difference (95% CI)	p Value
Sample size justification					
Reported	45 (75.0)	18 (64.3)	27 (84.4)	20.1 (−1.7 to 41.8)	0.073
Item 1					
Level of significance	39 (65.0)	16 (57.1)	23 (71.8)	14.7 (−9.3 to 38.8)	0.233
Item 2					
Power	45 (75.0)	18 (64.3)	27 (84.4)	20.1 (−1.7 to 41.8)	0.073
Item 3					
Treatment effect†	33 (55.0)	15 (53.6)	18 (56.3)	2.7 (−22.6 to 27.9)	0.835
Item 4					
Consistency with primary outcome	38 (63.3)	14 (50.0)	24 (75.0)	25.0 (1.2 to 48.8)	0.045
Item 5					
Allowance for attrition	18 (30.0)	7 (25.0)	11 (34.4)	9.4 (−13.6 to 32.4)	0.429
Item 6					
Number of clusters	58 (96.7)	27 (96.4)	31 (96.9)	0.4 (−8.7 to 9.6)	0.923
Median cluster size	39 (65.0)	15 (53.6)	24 (75.0)	21.4 (−2.4 to 45.2)	0.083
Item 7					
Variation in cluster size*	6 (10.0)	1 (3.6)	5 (15.6)	12.1 (−2.3 to 26.4)	0.201
Item 8					
Variation in outcome across clusters (ie, ICC)	33 (55.0)	11 (39.3)	22 (68.8)	29.5 (5.3 to 53.7)	0.022
Item 9					
Uncertainty of ICC (or equivalent)*	8 (13.3)	3 (10.7)	5 (15.6)	4.9 (−12.1 to 21.9)	0.712
All ItemS					
Number items reported median (IQR)	5.0 (2.5–6.0)	4.0 (1.0–6.0)	6.0 (5.0–6.0)	1.22 (0.07 to 2.36)	0.067
Reporting all nine items	0 (0)	0 (0)	0 (0)		

Values are numbers (percentages) unless stated. p Value is for the comparison of 1987–2012 publications and 2013–2014 publications using a χ^2^ test for proportions (categorical outcomes) or Mann-Whitney U test (where medians are reported), or (*) using Fisher's exact test.

†A sufficient reporting of the treatment effect consists of either a standardised effect size; a mean difference and SD; means in both arms and SD; proportions in both arms; proportion in one arm and a difference.

ICC, intracluster correlation.

Almost all trials reported the number of steps (90%), or this was deducible (98.3%), and 93.3% reported the number of clusters randomised per step (table 4). Many studies reported a schematic representation of the design (76.7%). However, only 26.7% of the trials explicitly reported whether the trial design was cross-sectional or cohort in nature; this increased to 71.7% when we made use of other reported items to deduce the type of design. In about 50% of the studies, it was unclear whether the cluster size reported in the sample size calculation related to the cluster size per measurement period or the total cluster size.

**Table 4:** Reporting of stepped-wedge cluster randomised trial sample size elements according to the proposed modification to the Cluster 2012 extension for cluster randomised trials

	All reports N=60	1987–2012 N=28	2013–2014 N=32	Absolute difference (95% CI)	p Value
Number of steps					
Explicitly reported	54 (90.0)	23 (82.1)	31 (96.9)	14.7 (−0.7 to 30.1)	0.058
Reported or deducible	59 (98.3)	27 (96.4)	32 (100.0)	3.6 (−3.3 to 10.4)	0.281
Number clusters randomised per step					
Reported	56 (93.3)	25 (89.3)	31 (96.9)	7.6 (−5.4 to 20.5)	0.240
Schematic representation					
Reported	46 (76.7)	20 (71.4)	26 (81.3)	9.8 (−11.7 to 31.3)	0.370
Design type (ie, cross-sectional/cohort)					
Explicitly reported	16 (26.7)	6 (21.4)	10 (31.3)	9.8 (−12.3 to 31.9)	0.391
Reported or deducible	43 (71.7)	19 (67.9)	24 (75.0)	7.1 (−15.8 to 30.0)	0.540
Clarity of cluster size†					
Total cluster size reported	17 (28.3)	8 (28.6)	9 (28.1)	−0.4 (−23.3 to 22.4)	0.969
Cluster size per measurement period reported	25 (41.7)	10 (35.7)	15 (46.9)	11.2 (−13.6 to 35.9)	0.382
Unclear/not reported	29 (48.3)	15 (53.6)	14 (43.8)	−9.8 (−35.1 to 15.4)	0.448

Values are numbers (percentages) unless stated otherwise.

p Value is for the comparison of 1987–2012 publications and 2013–2014 publications using a χ^2^ test for proportions.

†Some studies reported both total cluster size and cluster size per measurement period.

Our methodological assessment revealed that the majority (73.3%) allowed for clustering within the sample size calculation, but that only 33.3% allowed for time effects within the sample size calculation (table 5). Approximately 30% of the studies used the Hussey and Hughes methodology, with a small number using different methods which still allow for time effects. Fourteen (31.1%) of the studies reported using a methodology which clearly did not allow for time effects and a substantial number of studies (33.3%) did not report which methodology they used. Few studies incorporated additional design features into their power calculation (such as extended pre and post periods). There was an increase over time in the percentage of studies allowing for time effects from 16.7% pre-2012 to 44.4% post-2013 (p 0.063).

**Table 5:** Methodological assessment sample size calculations and trial justification in SW-CRTs, among those studies reporting a sample size calculation

	All reports N=45	1987–2012 N=18	2013–2014 N=27	Absolute difference (95% CI)	p Value
Allowance for clustering					
Number (%)	33 (73.3)	11 (61.1)	22 (81.5)	20.4 (−6.5 to 47.2)	0.130
Allowance for time effects					
Number (%)*	15 (33.3)	3 (16.7)	12 (44.4)	27.8 (2.3 to 53.2)	0.063
Allowance for repeated measurements†*					
Number (%)	3/24 (12.5)	2/11 (18.2)	1/13 (7.7)	−10.5 (−37.5 to 16.5)	0.576
Power methodology					
Hussey and Hughes	14 (31.1)	3 (16.7)	11 (40.7)	24.1 (−1.2 to 49.4)	0.087
Other, allowing for time effects*	2 (4.4)	0 (0)	2 (7.4)	7.4 (−2.5 to 17.3)	0.509
Other, not allowing for time effects	14 (31.1)	10 (55.6)	4 (14.8)	−40.7 (−67.3 to −14.2)	0.004
Not stated	15 (33.3)	5 (27.8)	10 (37.0)	9.3 (−18.3 to 36.8)	0.519
Power methodology for additional features					
Transition periods	0 (0)	0 (0)	0 (0)		
Interactions (eg, lag effects)	0 (0)	0 (0)	0 (0)		
Extended correlations*	2 (4.4)	2 (11.1)	0 (0)	−11.1 (−25.6 to 3.4)	0.155
Varying cluster size*	3 (6.7)	1 (5.6)	2 (7.4)	1.9 (−12.6 to 16.3)	1.000
Variation in outcomes across clusters‡					
Reported using ICC	20/33 (60.6)	8/11 (72.7)	12/22 (54.6)	−18.2 (−51.7 to 15.4)	0.314
Reported using CV*	10/33 (30.3)	2/11 (18.2)	8/22 (36.4)	18.2 (−12.2 to 48.6)	0.430
Reported using DE*	1/33 (3.0)	1/11 (9.1)	0/22 (0)	−9.1 (−26.1 to 7.9)	0.333
Reported using between cluster variation*	2/33 (6.1)	0/11 (0)	2/22 (9.1)	9.1 (−2.9 to 21.1)	0.542

Values are numbers (percentages) unless stated otherwise by year of publication.

p Value is for the comparison of 1987–1990 publications and 2013–2014 publications using a χ^2^ test for proportions or (*) using Fisher's exact test.

†Among those with a cohort design.

‡As a percentage of studies for which some measure of variation was reported.

CV, coefficient of variation; DE, design effect; ICC, intracluster correlation; SW-CRT, stepped-wedge cluster randomised trial.

## Discussion

We have carried out a methodological review to assess the quality of reporting and methodological rigour of sample size calculations in SW-CRTs. Of particular note, less than half of the trials in our review allowed for the temporal nature of the design in the power or sample size calculation. We also found that few studies acknowledged any repeated measures on the same individuals—yet, the majority of studies used a cohort design. Related to this, few studies explicitly described whether the study was cross-sectional or cohort in design, and in many studies, there was lack of clarity over whether the cluster size used in the sample size calculation was the total cluster size or the cluster size per measurement period.

It is known that lack of allowance for time effects in the sample size calculation for a SW-CRT can result in either an underpowered or overpowered trial.^13^ Early users of the parallel cluster trial failed to realise that sample size calculations required allowances for clustering, and this resulted in decades of underpower trials.^21^ Identification of similar oversights in the design of SW-CRTs at a time when they are just beginning to experience an upsurge in popularity might prevent similar years of poor practice. Furthermore, improvement with respect to clarity of reporting trials as cohort or cross-sectional would be a simple but important first step. Identification of these areas of poor performance can be used in the initial phase of the Delphi consensus study as potential items for inclusion in the CONSORT extension.^21^

We found that studies almost always reported the number of steps and the number of clusters randomised at each step, and a large majority provided a schematic representation of the trial design. Furthermore, many allowed for clustering. We observed some indication that the quality of reporting and methodological rigour improved in studies published after the publication on the most recent CONSORT extension for cluster trials. Many of the recent studies published were protocols, and so some of this improvement in quality might be attributable to the type of report (protocol or full report).^28^ However, in our analysis (not shown), we found little evidence of a difference between protocols and full reports.

The majority of studies used the Hussey and Hughes method to compute the power. The methodology proposed by Hussey and Hughes has some limitations. First, it does not immediately allow repeated measures on the same individual—yet, over half of the studies involved repeated measures using a cohort or open cohort design. Second, it assumes normality; whereas no previous studies have examined its application in the case of deviation from normality, more than half of the studies had a categorical primary outcome. Further methodological work is required to address these issues.

Recent methodological developments do now mean it is possible to determine the cluster size given other fixed design constraints, determine the number of clusters or number of steps needed^13^ Furthermore, a user-friendly sample size calculator to carry out these calculations has been developed and is available as an add-on function in the Stata statistical package.^29^ While some SW-CRTs might be very pragmatic and sample size determined by the number of observations available, properly designed evaluations should include a robust justification for the sample size. In implementation research and the evaluation of service delivery interventions, this will be necessary to justify funding resources needed to undertake the evaluation.

### Implications

Whereas sample size calculations that do not allow for the effect of clustering are likely to lead to underpowered SW-CRTs, those that do not allow for the effect of time might lead to studies being either underpowered or overpowered.^13^ For the case where the ICC is low, designing a SW-CRT using methodology for a parallel study is likely to lead to an underpowered study. Absolute differences in magnitude of power might be fairly low, for example, in the region of 10%. However, when the ICC is higher, designing a SW-CRT using methodology for a parallel design is bound to lead to an overpowered design at the expense of including vast numbers of observations which may contribute little to the power.^13^ Less work has been carried out on the drawbacks of powering a cohort SW-CRT as if it were of a cross-sectional design. However, repeated measures taken on the same individuals are likely to induce a reduction in variance of the treatment effect, and so lead again to a larger sample size than needed. Larger sample sizes than needed have important ethical implications. Bias in estimates of treatment effects is viewed by many to be more important than any lack of precision, and biases will not be prevalent unless the study did not take into account the time effects at the analysis stage. Whether or not allowances for time effects at the analysis stage are more frequent than at the design stage is not something we have considered, though it would seem unlikely that these mistakes are rectified if they are missed at the design stage.

Researchers need to specify estimates of ICCs in advance—as with any other cluster trial. While allowance for time effects are needed in the power calculation, these do not require any judgements or estimations, but are simply based on setting the number of steps, the number of clusters randomised per step and the average size of the cluster per step. When repeated measures are taken on the same individuals, then some specification will be needed on the strength of the correlation within individuals over time.^26^ However, sample size methods for SW-CRTs with repeated measures on the same individuals are yet to be developed.

### How our review differs from those already carried out

Other systematic reviews have been conducted to assess quantity and breadth of SW-CRTs.^4^ ^5^ ^7^ None of these reviews systematically assessed quality of reporting against the existing CONSORT statements and none assessed methodological rigour of sample size calculations. Our assessment of quality of reporting of sample size elements and of the methodological rigour of sample size calculations highlighted areas of poor performance. Identification of these areas of poor performance early in the use of this design will help mitigate these poor practices becoming common practices.

In previous reviews assessing quality of reporting in parallel cluster trials, assessments have been made against a smaller number of items recommended in the 2004 extension to the CONSORT statement to cluster trials.^23^ Here, we have made assessments against the 2012 extension to the CONSORT statement to cluster trials, even though many trials were designed before this reporting guideline was published. This is because our primary motivation was not to assess adherence to guidelines, but assess quality of reporting. Publication of CONSORT statements have been found to be associated with limited increases in the quality of reporting, and our findings are consistent with this.^30–32^ Interestingly, the most notable temporal trends we observed were for CONSORT and CONSORT cluster items and not stepped-wedge items.

### Study limitations

We assessed reporting according to the CONSORT guidelines for individually randomised trials and the extension for CRTs. Some of these reporting items do not extend naturally to the stepped-wedge design. For example, the CONSORT statement for RCTs recommends that authors report whether attrition has been taken into account in the sample size calculation. However, SW-CRTs are often used in the evaluation of service delivery interventions and implementation research where outcomes are routinely collected and attrition is unlikely to be an issue. Similarly, in cross-sectional designs (15% of our sample), attrition is unlikely to be an issue. Trials not reporting acknowledgement of attrition might do so simply because it is not relevant.

We attempted to assess methodological rigour of the published sample size calculations. We did this by extracting information on the methodology cited for the sample size or power calculation; we did not replicate these calculations. However, we were able to ascertain how many studies seem to use the appropriate methodology. The vast majority of the trials were published before the design effect for SW-CRTs was established^12^—indeed, only one study used this approach.

## Conclusions

As expected, the quality of reporting of sample size calculations in SW-CRTs is suboptimal, and although there has been some improvement over time, a significant number of studies are not clearly identifying whether the study used a cross-sectional or cohort design; less than half allowed for the temporal nature of the design in the power calculation; and few acknowledged any repeated measures on the same individuals. This means that the majority of studies are not using a sample size methodology that matches the study design. While there is a need for further methodological development, we have identified specific areas for improvement that are relatively easy for authors to address. These areas of poor quality can be used as initial items to go forward into the consensus agreement needed for the guidelines of reporting for SW-CRTs.
